# Prolonged Response to Dabrafenib/Trametinib in Grade 3 Metastatic Pancreatic Neuroendocrine Tumor (NET G3) with BRAF V600E Mutation

**DOI:** 10.1007/s12029-024-01072-0

**Published:** 2024-05-30

**Authors:** Benjamin E. Ueberroth, Christopher H. Lieu, Robert W. Lentz

**Affiliations:** Division of Medical Oncology, Department of Medicine, University of Colorado School of Medicine, 12801 E 17th Ave, MS 8117, Aurora, CO 80045 USA

**Keywords:** Neuroendocrine, Carcinoid, Targeted therapy, Gastrointestinal, *BRAF*

## Abstract

**Purpose:**

Treatment of metastatic pancreatic neuroendocrine tumors (pancNETs), particularly grade 2 (G2) and grade 3 (G3), often presents a dilemma in choosing from multiple similarly efficacious therapies. Data on targeted therapies for these tumor types is limited, and this report presents BRAF-targeted therapy as a therapeutic option for metastatic pancNET G3.

**Methods:**

This is a case report of a patient with G3 pancNET metastatic to the liver, lung, lymph node, and scalp (soft tissue) treated with dabrafenib/trametinib (D/T) in the presence of a BRAF V600E mutation detected in tumor tissue.

**Results:**

This patient has demonstrated an ongoing partial response to therapy at all involved sites for nearly 15 months with minimal side effects attributable to D/T.

**Conclusion:**

Dabrafenib/trametinib therapy for BRAF-mutated metastatic pancNETs provides a novel treatment option and, especially in the G3 setting, should be considered a first-line option. Tumor testing for actionable mutations should be undertaken at the time of diagnosis and/or progression to identify novel therapeutic avenues in these rare tumors.

## Introduction

Treatment options for unresectable pancreatic neuroendocrine tumors (pancNETs) are diverse, including somatostatin analogues (SSAs), peptide receptor radionuclide therapy (PRRT), small molecular inhibitors, and systemic chemotherapy [1]. Since the updated World Health Organization (WHO) grading system in 2019, well-differentiated, grade 3 (G3) NETs, defined by a Ki-67 greater than 20%, present a treatment dilemma with limited supporting data. Unlike poorly differentiated neuroendocrine carcinoma, G3 well-differentiated NET often does not respond to platinum plus etoposide (unless Ki-67 is quite high), and unlike well-differentiated G1-2 NET, G3 NET often does not express somatostatin receptors rendering SSAs and PRRT ineffective [2]. Further, PRRT is only FDA-approved for G1-2 NET [3]. The incidence of gastroenteropancreatic neuroendocrine neoplasms (GEP-NENs) is on the rise with the most recent analyses suggesting 3.5–4 cases per 100,000. G3 NETs are estimated to make up 5–10% of these cases [4, 5].

Tumor molecular profiling has become routine in other malignancies. Specifically, the *BRAF* V600E mutation has been identified as a relatively prevalent mutation across multiple solid organ tumors, leading to FDA accelerated approval for dual *BRAF*/*MEK* inhibitor therapy in any solid organ malignancy once progressed through standard therapies [6]. Response to these therapies in diseases ranging from melanoma to colorectal carcinoma is well-established [7, 8]. We present the first report of a significant, durable response to dabrafenib/trametinib (D/T) therapy in a patient with G3 pancNET harboring a *BRAF* V600E mutation who progressed through prior systemic therapy. The patient provided informed consent to publish this case report.

## Case Report

**Fig. 1:**
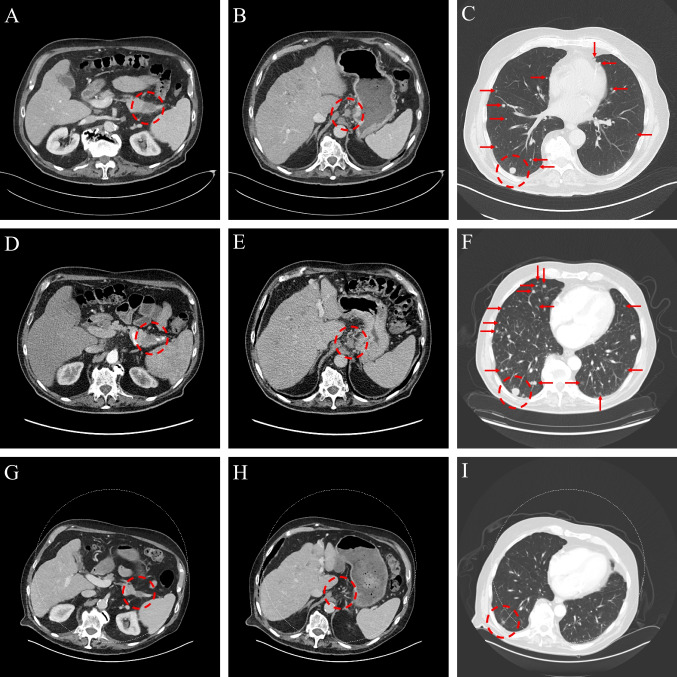
Images of primary pancreatic tumor, gastrohepatic lymph node metastasis, and pulmonary metastases at diagnosis, immediately before dabrafenib/trametinib (D/T) initiation, and after 14.5 months of D/T therapy. **A** Primary pancreatic neuroendocrine tumor at diagnosis (28 × 16 mm). **B** Gastrohepatic lymph node metastasis at diagnosis (15 × 11 mm). **C** Pulmonary metastases at diagnosis (index lesion 11 × 10 mm). **D** Primary pancreatic neuroendocrine tumor before D/T initiation (31 × 12 mm). **E** Gastrohepatic lymph node metastasis before D/T initiation (19 × 13 mm). **F** Pulmonary metastases before D/T initiation (index lesion 16 × 15 mm). **G** Primary pancreatic neuroendocrine tumor after 14.5 months of D/T (17 × 12 mm). **H** Gastrohepatic lymph node metastasis after 14.5 months of D/T (unmeasurable). **I** Pulmonary metastases after 14.5 months of D/T (index lesion 8 × 6 mm)

An 87-year-old man with background type 2 diabetes mellitus, cryptogenic cirrhosis, and benign prostatic hypertrophy presented with intermittent postprandial vomiting, 15–20 lbs (7–9 kg) weight loss, and fatigue. Baseline imaging showed a pancreatic tail mass (Fig. 1A) and metastatic disease involving the liver (Fig. 1B), lymph nodes (Fig. 1C), lungs (Fig. 1D), and left occipital scalp. A biopsy of a liver lesion confirmed a metastatic well-differentiated grade 3 (Ki-67 index of 37%, per WHO criteria) pancNET. Pretreatment Gallium-68-DOTATATE PET/CT showed only minimal DOTATATE avidity in sites of disease, which was felt to be insufficient to benefit from SSA or PRRT. Pre-treatment chromogranin A (CgA) was 155 ng/ml (normal, 0–103 ng/ml) and proved to be an unreliable tumor marker during subsequent treatment. After discussing multiple treatment options, capecitabine/temozolomide (CapeTem) was selected as the initial therapy.

After four cycles of CapeTem, interval imaging showed enlargement of the liver (Fig. 1F), nodal (Fig. 1G), and lung metastases (Fig. 1H), and palpable growth of the left occipital scalp nodule (the latter was biopsied and consistent with known NET). At the time of diagnosis, tumor tissue was sent for molecular testing (Tempus, Chicago, IL). Results showed a *BRAF* V600E missense mutation (c.1799 T > A, variant allele fraction 13.4%), NFE2L2 missense variant (c.101G > A, variant allele fraction 14.9%), microsatellite stable, and tumor mutational burden 3.7 m/MB. Variants of unknown significance were identified in *KMT2D*, *ASXL1*, and *SLC9A3R1.* Based on this result, *BRAF/MEK* inhibition was selected as second-line therapy after discussing multiple options. Of note, circulating tumor DNA testing (Guardant Health, Palo Alto, CA) was drawn after 8 months of D/T and did not show a *BRAF* mutation.

Within 2 weeks of starting D/T, the left scalp cutaneous metastasis had already decreased in size and totally disappeared by 1 month. The first interval abdominal MRI after two cycles of D/T showed an interval decrease in the size and number of liver metastases, as well as a change in appearance to predominantly hypoenhancing liver lesions (previously lesions all had arterial enhancement). An index liver lesion decreased from 25 to 17 mm in short axis (32% decrease). CT scans of the chest, abdomen, and pelvis at the same interval showed a significant decrease in the size and number of pulmonary nodules from more than 50 nodules before treatment to less than 10. There was also resolution of gastrohepatic ligament lymphadenopathy and regression of periportal and upper abdominal lymphadenopathy. No new or enlarging lesions were identified. At the time of publication, the patient remains on D/T therapy for over 14 months. Updated imaging at 14.5 months of D/T therapy was obtained showing ongoing response, with the index liver lesion further decreased in size to 14 mm in short axis (Fig. 1J), decreasing pulmonary nodules (Fig. 1L), hypoenhancement of the pancreatic tail lesion with a slight decrease in size (Fig. 1I), and resolution of gastrohepatic lymphadenopathy (Fig. 1K).

The patient has tolerated D/T therapy very well with minimal symptoms directly attributable to D/T. The patient experienced intermittent hypophosphatemia (grade 3 at nadir) attributed to D/T therapy which was corrected with oral phosphate repletion. He experienced intermittent fevers and chills, a common potential side effect of D/T, for which D/T was temporarily held [9]. Ultimately, all episodes of fevers and chills were attributed to recurrent urinary tract infections (treated with antibiotics) rather than D/T. At his last clinic visit, the patient was without anorexia, nausea, vomiting, diarrhea, or skin rash. He has not required any D/T dose adjustments.

## Discussion

Optimal first-line therapy and treatment sequencing are subjects of ongoing study in patients with unresectable, metastatic pancNETs, particularly in well-differentiated G3 tumors [3, 5]. Treatment choices can be more limited in patients such as this with low (or no) uptake on somatostatin receptor imaging, limiting the potential efficacy of SSA therapy and PRRT. Consensus guidelines for G3 NET recommend enrollment in a clinical trial as the preferred treatment [10].

Existing therapies for G3 NET generally demonstrate a progression-free survival (PFS) of around 1 year and stability tends to dominate the response landscape over tumor regression. CapeTem has perhaps the most inspiring data specific to GEP-NET G3, demonstrating a median PFS of 14.1 months but still only a 22.5% objective response rate (ORR) with the predominant minority of G3 patients having stable disease (42.5%) [11]. Another study examining all therapies utilized across a cohort of GEP-NET G3 showed a median PFS of 9.4 months for CapeTem and ORR of 35% [3]. In a mixed cohort of NET G3 and NEC, there was no significant difference in PFS, OS, or ORR between CapeTem and cisplatin/etoposide (EP), with a PFS of 12.6 months for CapeTem and 13.6 months for EP [12]. Although studies specifically in pancNET G3 are limited, FOLFOX carries a median PFS of 6.9 and 13.0 months and ORR of 28.6 and 56.4% across two small retrospective cohorts, respectively [3, 13]. The potential toxicities and intravenous administration of FOLFOX, as well as EP regimens, generally limit their use in NET G3 where other therapies are available with a lower rate of adverse effects. Everolimus lacks response data specific to NET G3 but in a large cohort of low- and intermediate-grade pancNETs, everolimus demonstrated a median PFS of 11.0 months compared to 4.6 months with placebo [14]. Sunitinib was studied across all GEP-NENs and demonstrated a median PFS of 11 months across all grades; the ten patients with NET G3 had a median PFS of 6.0 months [15]. Dual immune checkpoint inhibitor therapy has been studied in two mixed cohorts of NET G3 and NEC. With ipilimumab/nivolumab, ORR was 20% but responses were only seen in NEC patients [16]. With durvalumab/tremelimumab in a mixed NEC and G3 NET cohort, median PFS was 2.4 months [17].

In one of the largest tumor-agnostic studies, *BRAF/MEK* inhibition with D/T was shown to have a median PFS of 11.4 months, ORR of 38%, and median duration of response of 25.1 months [18]. *BRAF* mutations have been identified in diverse tumors ranging from melanoma to hematologic malignancies [19]. Anaplastic thyroid cancer (ATC) is another malignancy that harbors *BRAF* mutations in 25–45% of cases, and *BRAF*-targeted therapies have shown efficacy in this aggressive malignancy with a PFS around 6 months in a recent meta-analysis [20, 21]. Melanoma is another solid organ malignancy with relatively high rates of *BRAF* mutation, estimated to occur in about 50% of cases [22]. Most studies show a median PFS of about 12 months [23]. The 14.5-month ongoing response discussed in this case is thus already at least as good, if not outperforming, the PFS estimates in *BRAF*-mutated NETs, as well as other solid organ tumors like ATC and melanoma. The V600E mutation is the most common mutation seen across all malignancies, which leads to constitutive phosphorylation and thereby activation of the protein kinase. Acquired resistance to V600E inhibitors (e.g., dabrafenib) has led to the discovery of the *MEK-ERK* pathway in circumventing *BRAF* V600E inhibition. Development of the *MEK* inhibitor trametinib has increased response rates with dual D/T therapy over dabrafenib alone, but resistance mechanisms still develop, leading to eventual progression in most malignancies [19].

The largest cohorts of GEP-NEN molecular testing, which unfortunately often lack specific WHO grading beyond “high” and “low” grade, show that *BRAF* mutations occur between 5 and 15% of the time in GEP-NENs [24, 25]. Other cohorts specific to well-differentiated GEP-NENs vary, with one cohort documenting a *BRAF* mutation in 1/69 patients (1.4%) and another in 6/80 patients (7.5%) [26, 27]. The first cohort did not contain any pancNETs, only small bowel and rectal, perhaps suggesting lower rates of *BRAF* mutation in NETs originating from those sites compared to the pancreas [27]. Furthermore, the lower rates of *BRAF* mutations in some of these small cohorts of well-differentiated GEP-NENs suggest that *BRAF* mutations may be more frequent in NEC compared to lower-grade NETs of GI or pancreas origin [28, 29]. This may suggest that *BRAF* mutation is more common in more aggressive GEP-NENs such as NET G3 or NEC. As alluded to above, literature that predates the most recent WHO grading often lumps NET G3 and NEC together as “high-grade” NENs, limiting the interpretation of some of these studies as it pertains to the role of *BRAF* in the aggressiveness of the disease. Further study is thus warranted on the rates of *BRAF* mutation in various grades of NET as well as NEC, and if variances in these findings are clinically relevant to predicting disease behavior beyond known factors such as Ki-67.

In view of the likely similar efficacy and improved tolerability of D/T (with *BRAF* V600E mutation) compared to other treatment options for G3 NET (acknowledging limitations of cross-trial comparisons), and the possibility of identifying a targetable alteration, routine molecular testing of pancNETs, and all GEP-NENs, should be pursued. Even if other therapies are chosen in the first or second lines, potentially actionable mutations may be identified allowing for prolonged treatment and disease control in subsequent lines of therapy. Specifically for G3 NETs, *BRAF/MEK* inhibition should be considered as first-line therapy. There are occasional case reports of targeted treatment of *BRAF* V600E mutated pancNETs as well as poorly differentiated neuroendocrine carcinoma (NEC) of GI origin with varying results; however, this is the first report of D/T treatment specific to NET G3 of pancreatic origin [26, 30, 31].

While this case focuses on the utility of *BRAF*-targeted therapies in NET, other mutations may be identified if molecular testing of NETs becomes routine. Specifically, targeted therapies for mutations in *KRAS*, *BRCA1/2*, *ATM*, *NTRK*, *FGFR*, and *RET* merit consideration after progression on early-line therapies when those mutations are present. Many of these mutations have been identified in small numbers in the aforementioned cohorts [28, 29]. Data is limited on implementing targeted therapies in this disease. Further study is warranted to better define the molecular landscape of potentially targetable alterations in GEP-NENs, response to targeted therapies, and integration of these drugs into existing treatment paradigms.

## Conclusion

Dual *BRAF/MEK* inhibition with dabrafenib/trametinib therapy led to a significant and durable partial response in a treatment-refractory patient with unresectable, metastatic well-differentiated pancreatic neuroendocrine tumor grade 3. Molecular testing of NET of gastrointestinal and pancreatic origin should be routinely considered to expand potential treatment options.

## Data Availability

No datasets were generated or analysed during the current study.
